# Genome-Wide Identification and Expression Profiling of Pyruvate Kinase Genes in Litchi Under Calcium-Magnesium Foliar Treatment

**DOI:** 10.3390/plants14172764

**Published:** 2025-09-04

**Authors:** Muhammad Sajjad, Jiabing Jiao, Hassam Tahir, Ling Wei, Wuqiang Ma, Muhammad Zeeshan Ul Haq, Muhammad Amir Farooq, Kaibing Zhou

**Affiliations:** 1 School of Breeding and Multiplication (Sanya Institute of Breeding and Multiplication), School of Tropical Agriculture and Forestry, Hainan University, Sanya 572025, China; drmuhammadsajjad@hainanu.edu.cn (M.S.); drzeeshanulhaq@gmail.com (M.Z.U.H.);; 2 School of Tropical Agriculture and Forestry, Hainan University, Haikou 570228, China; 3 College of Landscape and Horticulture, Yunnan Agricultural University, Kunming 650201, China

**Keywords:** pyruvate kinase (PK), calcium and magnesium (Ca+Mg), bioinformatics, hormonal regulation, gene expression

## Abstract

Pyruvate kinase (PK) is a key enzyme in glycolysis that regulates sugar metabolism and energy production, thereby influencing fruit quality. The ‘Feizixiao’ litchi, widely cultivated in Hainan Province, faces sugar reduction during fruit ripening. This study evaluated the effects of the foliar application of calcium and magnesium (Ca+Mg) during the fruit expansion stage to alleviate this problem. Ca+Mg foliar application significantly enhanced soluble sugar content, promoted peel coloration, and reduced respiration and PK activity. Genome-wide analysis identified 19 PK genes (*LcPKs*) exhibiting diverse exon-intron structures and conserved motifs. Phylogenetic analysis revealed both conserved and species-specific features, while subcellular localization predicted that most LcPK proteins are likely to be localized in the cytoplasm. Synteny analysis showed closer evolutionary relationships with species in the same genus than with *Arabidopsis*. Cis-regulatory element analysis implicated *LcPKs* in light response, hormone signaling, growth, and stress adaptation. Hormonal assays at 63 and 70 DAA after treatment revealed increased abscisic acid (ABA) and ethylene levels under Ca+Mg application. These hormonal changes correlated with the downregulation of *LcPK3*, *LcPK4*, *LcPK5*, *LcPK8*, and *LcPK15*, as confirmed by qRT-PCR, indicating negative regulation by ABA and ethylene. This regulatory mechanism likely contributes to overcoming sugar receding in litchi pulp. These findings offer insights into the regulation of sugar metabolism and strategies for enhancing fruit quality through the management of genes and nutrients.

## 1. Introduction

Litchi (*Litchi chinensis*), a tropical fruit tree from the Sapindaceae family, is extensively cultivated in Guangdong, Guangxi, Hainan, Taiwan, and Sichuan provinces [1]. The ‘Feizixiao’ litchi, prized for its aroma, texture, and sweetness, exhibits’ delayed degreening,’ where the peel coloration lags behind sugar accumulation, limiting full red pigmentation [2]. Conversely, once the fruit surface attains full redness, the sugar content in the pulp begins to decline, resulting in a deterioration of flavor and an increase in acidity. This phenomenon, known as “sugar receding,” significantly impacts the fruit’s commercial value as a fresh product [3]. Foliar nutrient treatment with a Ca-Mg mixed solution has been shown to effectively reduce the “sugar receding” issue in ‘Feizixiao’ litchi [4]. The treatment inhibits aerobic respiration through glycolysis, the TCA cycle, and the pentose phosphate pathway (PPP), enhances cytochrome respiration, and suppresses the cyanide-resistant pathway [4].

Pyruvate kinase (EC 2.7.1.40) catalyzes the final step of glycolysis, converting phosphoenolpyruvate (PEP) and adenosine diphosphate (ADP) into pyruvate and adenosine triphosphate (ATP) in an irreversible reaction [5]. The pyruvate produced is a glycolytic end product and regulates various metabolic pathways. Under anaerobic conditions, it can be converted into lactic acid or ethanol. Moreover, it undergoes decarboxylation in aerobic conditions to generate acetyl-CoA, a crucial step in the TCA cycle [6]. Thus, PK is essential for the cell’s energy metabolism [7]. PK enzyme occurs in plants as two isoforms: plastidic pyruvate kinase (PKp) and cytoplasmic pyruvate kinase (PKc), each localized to distinct tissues [8]. PKc plays a key role in regulating sink-source dynamics and controlling plant carbon and respiratory metabolism [9]. PKp is crucial for the breakdown of storage compounds in germinating seeds and for the synthesis of seed oils [10]. The PK gene family is relatively large, with the *Arabidopsis thaliana* genome containing 4 PKp and 10 PKc genes [11]. In comparison, *Solanum tuberosum* has been identified to have 5 PKc and 4 PKp genes [12]. In Gossypium, 33 genes (19 PKc and 14 PKp genes) are responsible for encoding the putative subunits of PK [13]. PK typically survives as a homotetramer but can also form various structures, including monomers, heterodimers, heterotetramers, and heterohexamers [14]. These PK genes, which encode different isozymes, collectively form a large gene family that participates in numerous biological and metabolic processes in plants [15]. Numerous studies have highlighted the importance of PK in the glycolysis pathway across various plant species, such as *A. thaliana*, *Oryza sativa*, and *Nicotiana tabacum*. In *Arabidopsis*, PKp plays a crucial role by effectively converting sugars into essential precursors, such as pyruvate, which are vital for various anabolic pathways supporting seed germination and reproduction [16]. Deleting the *PKp_1* gene reduces seed oil by 60%, while overexpressing *PKpb1* restores it completely, and *PKpb2* only partially recovers it, emphasizing the key role of PK in lipid synthesis [17]. During lipid biosynthesis initiation, PKc expression decreases while PKp expression increases, indicating a shift in carbon flux from cytosolic to plastid metabolism [18]. In cotton, GhPK6, a PKc gene expressed explicitly in fibers, is crucial in promoting fiber elongation [13]. The PK genes are also vital for rice growth and development. For instance, mutations in OsPK1 result in reduced expression levels, which adversely affect gibberellin synthesis and lead to imbalances with abscisic acid. This disruption also affects the synthesis, transport, and metabolism of carbohydrates, ultimately leading to dwarfism and panicle enclosure [19]. OsPK2 (OsPKa1) is vital for starch synthesis and grain filling in *O. sativa*, while reduced expression of OsPK3 and OsPK4 hampers grain filling and storage compound accumulation [20].

Ca and Mg are essential for plant growth and hormonal balance [21]. Phytohormones such as auxins, ABA, jasmonic acid (JA), cytokinins, and ethylene regulate fruit development [22]. Ca is a hormone pathway messenger, while Mg’s role remains unclear [23]. Hormones also affect Ca distribution, with imbalances causing disorders such as blossom end rot [24]. ABA and ethylene drive ripening; methyl jasmonate (MeJA), CKs, salicylic acid (SA), and gibberellins (GA) aid ripening, defense, and color [25]. This study aims to determine how foliar Ca and Mg treatments prevent “sugar receding” in ‘Feizixiao’ litchi pulp by inhibiting PK activity, a key enzyme in respiration. We will evaluate the effects of Ca+Mg on respiration, soluble sugar content, and peel coloration to assess fruit quality. Additionally, we hypothesize that hormones regulate PK gene expression, affecting this process. Furthermore, we will identify and characterize the PK gene family in litchi through genomic and bioinformatics analyses and examine its role in sugar metabolism. The findings will support nutrient management strategies to maintain sugar levels and improve fruit quality.

## 2. Materials and Methods

### 2.1. Experimental Setup, Field Treatment, and Sample Collection

On 16 April 2022, 10 mature ‘Feizixiao’ litchi trees were selected from Team 7’s orchard in Jinpai Farm, Lingao County, Hainan Province. The trees were healthy, with uniform growth and high yield potential. This orchard in a tropical monsoon zone has temperatures of 23–24 °C, 2175 h of sunshine annually, and 1100–1800 mm of rainfall. Key phenological events include flowering (February to March), fruit drop (April), fruit expansion (late April), and ripening (mid-May). The trees were divided into two groups: one with 0.3% CaCl_2_ and MgCl_2_ (Ca+Mg) and the other with clean water (CK). Treatment was applied 35 days after anthesis (DAA), marking the start of rapid fruit expansion. The design included single-plant plots with five replicates per treatment [26,27]. Field measurements were taken three times a week between 9:00 and 10:00 a.m. Fruits from the middle section of the canopy were selected for dynamic sampling. Five medium-sized fruits per tree were marked, and sampling continued until whole pericarp reddening at 70 DAA. At each time point (35, 42, 49, 56, 63, and 70 DAA), 30 fruits per tree were collected, frozen in liquid nitrogen, and stored at −80 °C for analysis.

### 2.2. Determination of Soluble Sugar, Peel Coloration, and Metabolic Activities

Soluble sugar content in the pulp was measured using anthrone colorimetry. A 1 g pulp was ground, treated in a boiling water bath for 10 min, then centrifuged and boiled again for 10 min. After cooling, anthrone was added to react with various sugars, producing a blue-green solution with maximum absorption at 620 nm. The sugar content was quantified by comparing absorbance to a standard curve [28]. The hue angle (h) of the fruit peel was measured using a portable colorimeter. Six points were chosen for measurement: four randomly selected on the fruit’s equator, and one each at the pedicel and apex. The average of these six measurements was then calculated and recorded as the fruit’s coloration index.

Respiration rates via the EMP pathway in litchi fruit pulp were measured using the Pictrip O_2_/CO_2_ headspace analyzer (CheckMate 3, Dansensor, Ringsted, Denmark), with results expressed as mL CO_2_·kg^−1^·h^−1^. Sodium fluoride (10 mmol) was used as an EMP pathway inhibitor [29]. Measurements were taken at approximately 10:00 a.m. under a constant ambient temperature of 28 °C. The process began by recording the total respiratory rate of the sample. The residual respiration rate of the EMP pathway was measured after vacuum infiltration of the inhibitor. The respiration rate specific to the EMP pathway was then calculated by subtracting the residual rate from the total respiratory rate [30]. The method for measuring PK enzyme activity was based on the procedure [31]. A 2 g litchi pulp was mixed with Tris-HCl buffer, ground, and centrifuged. The supernatant’s absorbance at 520 nm was measured using an enzyme-labeling instrument. Enzyme activity was determined by comparing the absorbance to a standard curve created with fructose as the reference.

### 2.3. Hormonal Changes Analyzed Using Enzyme-Linked Immunosorbent Assay (ELISA)

ELISA-based analysis was conducted to quantify the concentrations of these hormones in litchi pulp from treated and control fruits, with five biological replicates per period, using kits purchased from Shanghai Yuanju Biotechnology Center, Shanghai, China. A 50 mg pulp sample was homogenized in 9 mL PBS, then centrifuged for 15 min at 4000 rpm. Micro ELISA Stripplates were supplemented with standard solutions and supernatants. Standard wells contained 50 μL of standard, and sample wells had 10 μL of sample plus 40 μL of diluent. After adding 100 μL of HRP-conjugate reagent, plates were incubated for 60 min at 37 °C. Then, 50 μL of Chromogen Solutions A and B was added, followed by 50 μL of Stop Solution after 15 min, causing a color change from blue to yellow. Hormone concentrations were determined by measuring absorbance at 450 nm.

### 2.4. PK Gene Family Identification and 3D Protein Modeling

The protein sequence of the *PK* gene was blasted against the latest annotated litchi genome database (2024 version) using the BlastP program to identify its family members (available at http://www.sapindaceae.com/index.html [32], accessed on 10 August 2024). The PK protein domain was examined using the InterPro tool (IPR001697) (https://www.ebi.ac.uk/interpro, accessed on 12 August 2024). Using the approach described in [33], we evaluated the chromosome number (Chr), chromosomal position, protein molecular weight (PMW), grand average of hydropathicity (GRAVY), isoelectric point (pI), and coding sequence (CDS) length of these proteins to gain a deeper understanding. Furthermore, Phytozome V13 (https://phytozome-next.jgi.doe.gov/, accessed on 12 August 2024), provided the *Arabidopsis* genome. The ExPASy ProtParam program (protein parameters calculator) (https://www.web.expasy.org, accessed on 1 September 2024), and TBtools-II (Toolbox for Biologists, v2.33) were used to conduct these analyses.

Homology modeling was performed using SWISS-MODEL (https://swissmodel.expasy.org/, accessed on 3 September 2024), which searched the SWISS-MODEL Template Library (SMTL) using BLAST and HHblits [34]. Templates were selected based on GMQE scores, and 3D models were generated using ProMod3 v1.3.0. Functional predictions of the modeled proteins were conducted via the ProFunc server (https://www.ebi.ac.uk/thornton-srv/databases/profunc/, accessed on 3 September 2024) [35].

### 2.5. Analysis of Motif Identification, Domain, Gene Structure, and Phylogenetic Study

We used the National Center for Biotechnology Information (NCBI), CDD batch (Conserved Domains Database and Resources), MEME Suite (http://meme-suite.org/ (version 4.11.3), accessed on 5 September 2024), and the GFF3 file in TBtools-II to show the conserved motifs, domains, and gene structures of PK gene family. TBtools-II was used to examine the intron and exon design of the *LcPK* genes [36]. Phylogenetic analysis used PK protein sequences from *L. chinensis*, *A. thaliana*, *Solanum lycopersicum*, and *Malus × domestica*. The sequences were aligned using the MUSCLE method in MEGA 11, and a phylogenetic tree was constructed with the Maximum Likelihood approach and 1000 bootstrap replications to ensure accuracy. The resultant phylogenetic tree was shown using the iTOL software [37], accessed on 5 September 2024, offering a clear and thorough understanding of the evolutionary relationships between these species.

### 2.6. Ka/Ks Value Calculation, Chromosomal Location, and Subcellular Localization

MEGA-11 software calculated the nonsynonymous (Ka) and synonymous (Ks) substitution rates, as well as the Ka/Ks ratio, using the coding sequence (CDS) of the genome. TBtools-II (v.2.210.) was used to visualize the chromosomal positions of the 19 *LcPK* genes, as of 7 September 2024, which were obtained from the GFF3 file of the litchi genome. We utilized two online predictive programs, WoLF PSORT (https://www.genscript.com/wolf-psort.html, accessed on 8 September 2024) and CELLO (http://cello.life.nctu.edu.tw/, accessed on 8 September 2024), to predict the subcellular localization of members of the 19 LcPK gene family.

### 2.7. Gene Duplication, Synteny Analysis, and Cis-Acting Component Analysis

Collinearity analysis data between species were sourced from the Sapindaceae genomic database (http://www.sapindaceae.com/index.html, accessed on 10 September 2024), which contains a variety of species, including *L. chinensis*, *Dimocarpus longan*, *Nephelium lappaceum*, and *Acer yangbiense*, as well as Phytozome V13 (https://phytozome-next.jgi.doe.gov/, accessed on 12 August 2024), which includes *A. thaliana*. The chromosome distribution of the PK family was generated using MCScanX with default parameters, after retrieving the genome and GFF files of the required species [38,39]. The homology of the LcPK gene family between litchi and other plant species was subsequently analyzed using the dual synteny plotter in TBtools-II. The 2000 bp upstream region of the LcPK genes was obtained from the litchi genome and used as the promoter sequence to identify cis-acting elements, which were analyzed using the PlantCare program (http://bioinformatics.psb.ugent.be/webtools/plantcare/html, accessed on 15 September 2024) [40].

### 2.8. Expression Analysis of LcPK Genes

Transcriptome analysis was performed on litchi fruits at 35, 63, and 70 DAA from treated and control fruits. The expression of the 19 *LcPK* genes was computed using Fragment Per Kilobase Million (FPKM) values, and TBtools-II was used to create a heatmap.

### 2.9. qRT-PCR Analysis

The RNA was extracted from litchi pulp at 35, 63, and 70 DAA from treated and control fruits, with three biological replicates per period. The Illumina platform was used to create and sequence cDNA libraries following RNA purification. The following nine Differentially Expressed Genes (DEGs) were chosen for qRT-PCR validation: *LcPK2*, *LcPK3*, *LcPK4*, *LcPK5*, *LcPK8*, *LcPK10*, *LcPK11*, *LcPK12*, and *LcPK15*. Specific primers were designed using Primer3 and manufactured by Boshang Biotechnology (Shanghai, China). Following the manufacturer’s instructions, RNA was extracted from the pulp using the SteadyPure Plant RNA Extraction Kit (Hangzhou Aikerui Biotechnology Co., Ltd., Hangzhou, China) and then reverse-transcribed using the EvoM-MLV Reverse Transcription Premix Kit (Hangzhou Aikerui Biotechnology Co., Ltd., Hangzhou, China). On a qTOWER3 device (Jena, Germany), qRT-PCR validation was carried out using the 2× Q3 SYBR qPCR Master Mix (Universal) (TOLOBIO). The gene expression levels were determined using the 2^−ΔΔCT^ technique [41]. The specifics are listed in Table S1.

### 2.10. Statistical Analysis

Data analysis was performed using SAS 9.0, with ANOVA for variance analysis and Duncan’s multiple comparison method. A *t*-test was applied to compare the treatment and control groups (CK). Graphs were generated with GraphPad Prism 8.0.1, and a differential gene expression heatmap was created using TBtools-II [42].

## 3. Results

### 3.1. Effects of the Treatment on Soluble Sugar Content, Peel Coloration, and Metabolic Activities

**Figure 1:**
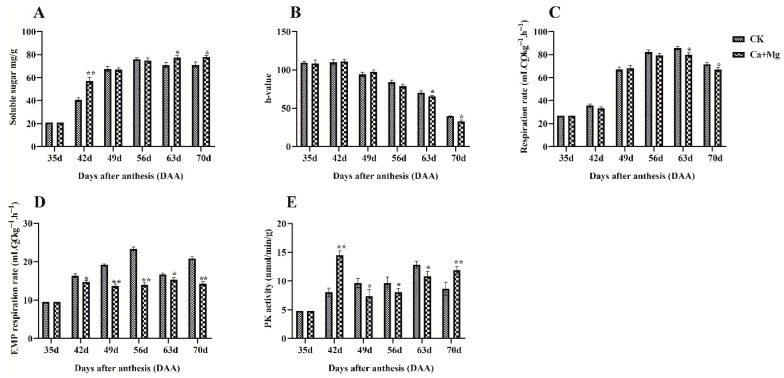
Effect of Ca+Mg treatment on sugar content, peel coloration, and metabolic activities: (**A**) soluble sugar content; (**B**) h-value; (**C**) respiration rate; (**D**) EMP pathway; and (**E**) PK activity. Significant differences between the Ca+Mg treatment and CK groups are indicated by * (*p* < 0.05) and ** (*p* < 0.01).

The effect of the treatment on the dynamic changes in soluble sugar content in pulp (Figure 1A) was assessed. The Ca+Mg treatment and the CK exhibited an overall increase in soluble sugar content. However, the treatment reached its peak sugar levels at 63 and 70 DAA. In contrast, the CK group showed a decline in sugar content at these time points, resulting in the “sugar receding” phenomenon. Notably, the treatment maintained elevated sugar levels at 63 and 70 DAA, effectively preventing the “sugar receding” effect. At 42, 63, and 70 DAA, the soluble sugar content in the treatment group was significantly higher than that in the control group. The effect of the treatment on peel coloration (Figure 1B). No significant changes were observed in either group until 56 DAA. After 49 DAA, the h-value of both the treatment and CK groups declined, indicating the onset of peel reddening. At 63 DAA, both groups exhibited visible reddening, but the treatment group had a higher h-value than CK, suggesting that coloration began earlier in the treatment group. By 70 DAA, the treatment group maintained a higher h-value, indicating that the treatment accelerated the coloration process, resulting in a redder peel than the control. The total respiration rate of litchi fruit under the Ca+Mg treatment was notably lower than that of the CK group at 63 and 70 DAA (Figure 1C). While the CK group caused a partial increase in the respiration rate, the Ca+Mg treatment inhibited it. A similar pattern was observed in the respiration rate through the EMP pathway in litchi pulp (Figure 1D). The Ca+Mg treatment consistently maintained a significantly lower respiration rate than the CK group from 42 to 70 DAA. Overall, the treatment reduced respiration through the EMP pathway, with the most pronounced effect in the Ca+Mg treatment. PK activity in litchi pulp also showed significant differences between the Ca+Mg treatment and the CK group (Figure 1E). At 49, 56, and 63 DAA, the Ca+Mg treatment resulted in significantly lower PK activity than the CK group, suggesting inhibition of the EMP pathway during these stages. However, at 42 and 70 DAA, PK activity in the CK group was significantly higher, indicating that the suppression of the EMP pathway had lessened at these times Points.

### 3.2. Genome-Wide Investigation and 3D Structures of the PK Gene Family

We identified 19 *PK* genes from the litchi genome through genome-wide analysis and named them (*LcPK1* to *LcPK19*). The ExPASy ProtParam server was used to derive the physicochemical properties of the study protein. It overviews their chemical and structural characteristics, including PMW, GRAVY, pI, CDS length, Chr number, and chromosomal position (Table 1). Amino acid length ranged from 78 (*LcPK12*) to 709 (*LcPK4*) AA, and CDS lengths ranged from 237 (*LcPK12*) to 2130 (*LcPK4*) bp. The PMW varied between 12 (*LcPK18*) and 86.1 (*LcPK12*) kDa. The GRAVY value measures protein hydrophilicity or hydrophobicity. Notably, all GRAVY values for the 19 *LcPK* genes ranged from −0.35 (*LcPK17*) to 0.072 (*LcPK3*). Proteins with negative GRAVY values are hydrophilic and more soluble, while positive values indicate hydrophobicity and lower solubility.

**Figure 2:**
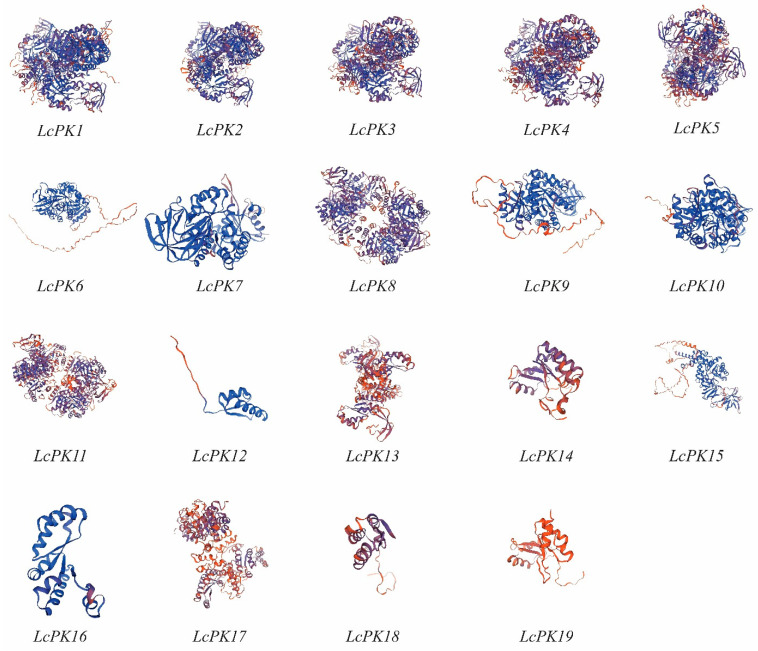
The 3D structures of 19 *LcPK* genes were modeled using SWISS-MODEL. α-helices, β-sheets, and coils are depicted as spirals, broad arrows, and thin loops, respectively. The protein backbone is shown in blue, and the orange and red regions highlight potentially functionally significant areas.

The 3D modeling links protein structure to function through homology searches in the Protein Data Bank. Using the SWISS-MODEL algorithm, we predicted the 3D structures of 19 LcPK proteins, with sequence identities ranging from 30.06% to 94.69% compared to template proteins (Figure 2). This variation confirmed the reliability of homology modeling, as a minimum identity of 30% was sufficient for accurate prediction. These results indicated that the proteins were suitable for structural.

### 3.3. Conserved Protein Motif Analysis, Gene Structure, and Phylogenetic Analysis

**Figure 3:**
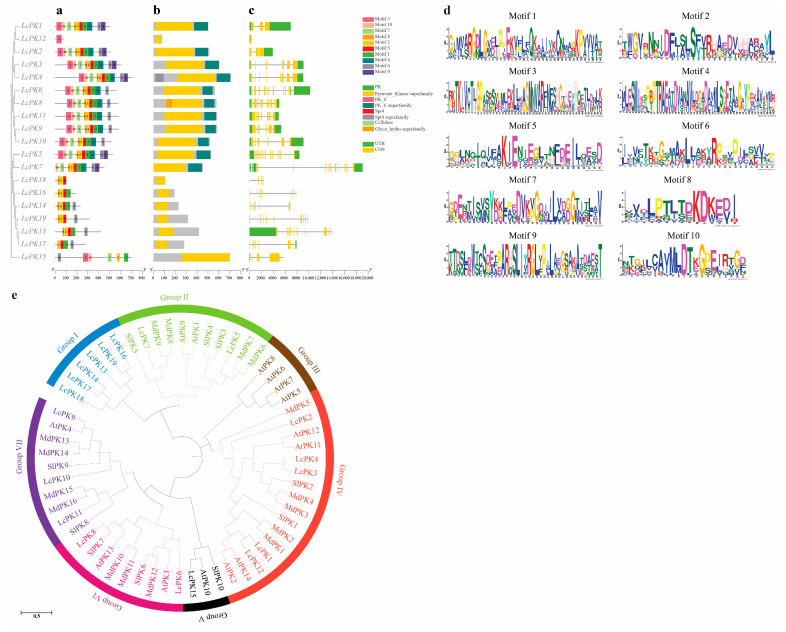
Comparative analysis of LcPK gene motifs, domains, and structures: (**a**) Boxes of different colors represent the 10 conserved motifs; (**b**) PK-family protein domains; (**c**) The intron-exon organization of *LcPK* genes is illustrated, with introns shown in green and exons (CDS) in yellow; (**d**) motif elements, and (**e**) A phylogenetic tree of the LcPK protein from *Litchi chinensis*, *A. thaliana*, *Solanum Lycopersicum*, and *Malus × domestica* was constructed using the Maximum Likelihood method with 1000 bootstrap replications in MEGA-11, with distinct groups represented by different colors. The scale bar refers to a phylogenetic distance of 0.5 amino acid substitutions per site.

MEME analysis identified 10 conserved motifs among the 19 LcPK proteins (Figure 3a). LcPK1–5 contained all motifs, while others showed varying patterns; for instance, LcPK12 had only motif 3, and LcPK18 and LcPK16 contained limited combinations. Most proteins within the cluster exhibited similar motif profiles, indicating functional or structural similarities. Domain analysis using the NCBI database confirmed that all LcPK proteins belong to the PK superfamily, and the domain structures were visualized with TBtools-II (Figure 3b). The structural analysis of 19 *LcPK* genes based on the litchi genome revealed variation in exon–intron organization (Figure 3c). Intron counts ranged from one to four, and exon counts from one to sixteen. LcPK1 and LcPK12 had three introns and three exons, LcPK6 and LcPK8 had three and twelve exons, and LcPK5 had two and sixteen exons. Some genes, such as LcPK2 and LcPK15, had only one intron, while others, like LcPK7, exhibited greater complexity with four introns and fourteen exons. This diversity suggests functional and structural differentiation among *LcPK* genes.

The phylogenetic tree of PK proteins, constructed using maximum-likelihood methods, reveals the evolutionary relationships among 59 PK proteins from four species: *L. chinensis* (19), *A. thaliana* (14), *S. lycopersicum* (10), and *Malus × domestica* (16), grouping them into seven distinct clusters (Figure 3e). Group I comprises six proteins unique to *L. chinensis*, indicating a possible species-specific expansion. Group II includes eleven proteins from all four species, suggesting evolutionary conservation and potential shared functions. Group III comprises four *A. thaliana* proteins, reflecting a lineage-specific grouping. Group IV is the largest, comprising sixteen proteins from all species, which highlights a highly conserved and potentially ancestral PK subgroup. Group V consists of three proteins: LcPK15, SlPK10, and AtPK10. Notably, LcPK15 and AtPK10 exhibit strong similarity, indicating functional conservation between litchi and *Arabidopsis*. Group VI comprises nine proteins, with a close relationship between LcPK8 and SlPK7, suggesting conserved roles between litchi and Solanum. Lastly, Group VII comprises ten proteins from all species, with LcPK9 being closely related to AtPK4, suggesting a possible evolutionary link and shared functional characteristics.

### 3.4. Ka/Ks Value Calculation, Chromosomal Location, Subcellular Distribution, and Gene Duplication Analysis

**Figure 4:**
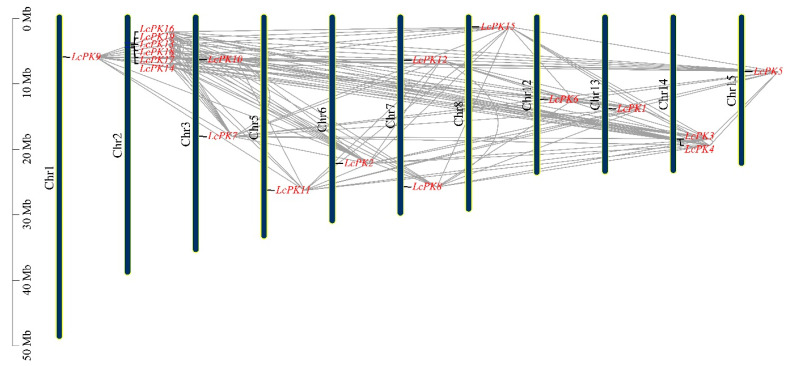
The scattering of 19 *LcPK* genes on 15 litchi chromosomes is shown, with genes in red and chromosomes in black.

Ka and Ks analysis reveals evolutionary processes and selection types in duplicated gene pairs. A Ka/Ks ratio < 1 suggests purifying selection, >1 suggests positive selection, and =1 suggests neutral selection. We analyzed Ka, Ks, and Ka/Ks ratios for 129 homologous LcPK gene pairs (Table S2). Our analysis revealed that most *LcPK* genes, with a Ka/Ks ratio below 1, indicate purifying selection. However, 12 gene pairs exhibited a Ka/Ks ratio greater than 1, indicating positive selection. To localize the 19 *LcPK* genes, we obtained chromosomal localization data and used TBtools II software (v.2.210.) to map and distribute all *LcPK* genes across the litchi genome. Each gene is represented in red while the chromosome is in black, corresponding to their periods, as illustrated in the phylogenetic tree (Figure 4). The mapping highlighted genetic variation in the scattering of these genes across 15 chromosomes, with Chromosome 2 (Chr2) featuring the most significant number, comprising six genes: *LcPK16*, *LcPK19*, *LcPK13*, *LcPK18*, *LcPK17*, and *LcPK14*. Chr3, 7, and 14 each contained two genes, while Chr1, 5, 6, 8, 12, 13, and 15 each had one gene.

**Figure 5:**
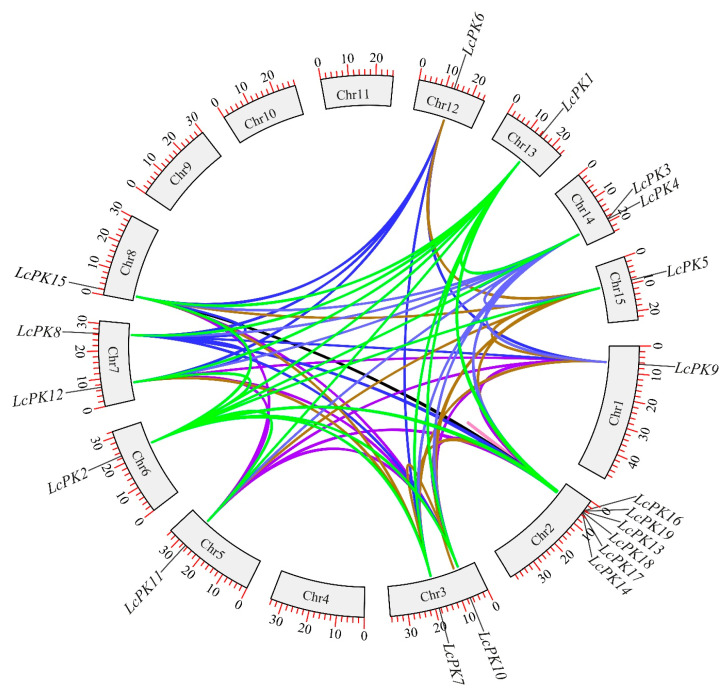
A circos plot of the *LcPK* gene duplication pair in *Litchi chinensis*. The various colors represent genes that developed through gene duplication.

The subcellular localization of the 19 *LcPK* genes was created using WoLF PSORT software and the CELLO server. Our results indicate that LcPK genes are found in various organelles, with the cytoplasm being the most common location, followed by the mitochondria, chloroplasts, nucleus, and extracellular space (Table S3). We discovered 129 pairs of duplicated gene couples among the 19 *LcPK* genes. To further understand the selection pattern of LcPKs, we found a 20-tandem duplication among all of these genes (Figure 5). Most of the genes were less than one, suggesting that the *LcPK* genes in these groups were exposed to purifying selection pressure.

### 3.5. Synteny Analysis and Cis-Acting Component Analysis

**Figure 6:**
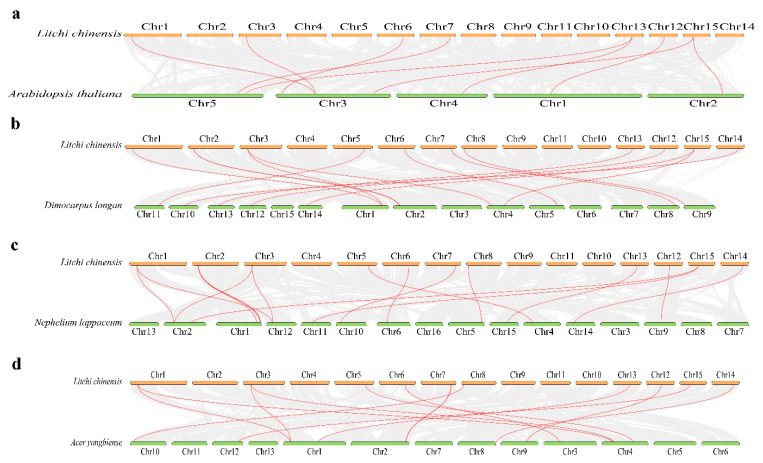
The synteny analysis of *LcPK* genes between *Litchi chinensis* and each of the four representative plant species is depicted in sections (**a**–**d**), where gray lines indicate adjacent genomic blocks and red lines highlight co-linear *LcPK* gene pairs.

To investigate the evolutionary relationships of LcPK genes in *L. chinensis*, we conducted a comparative analysis with three closely related species: *D. longan*, *N. lappaceum*, and *A. yangbiense*. *A. thaliana* was also included as a model organism for constructing the syntenic framework of the PK gene family. Our results revealed that 9 *LcPK* genes displayed co-linearity with *Arabidopsis*, 14 genes in *D. longan*, 17 in *N. lappaceum*, and 13 in *A. yangbiense* (Figure 6). In contrast, only 9 PK homologs were identified in the *Arabidopsis* genome, and *N. lappaceum* contained 17 homologs.

**Figure 7:**
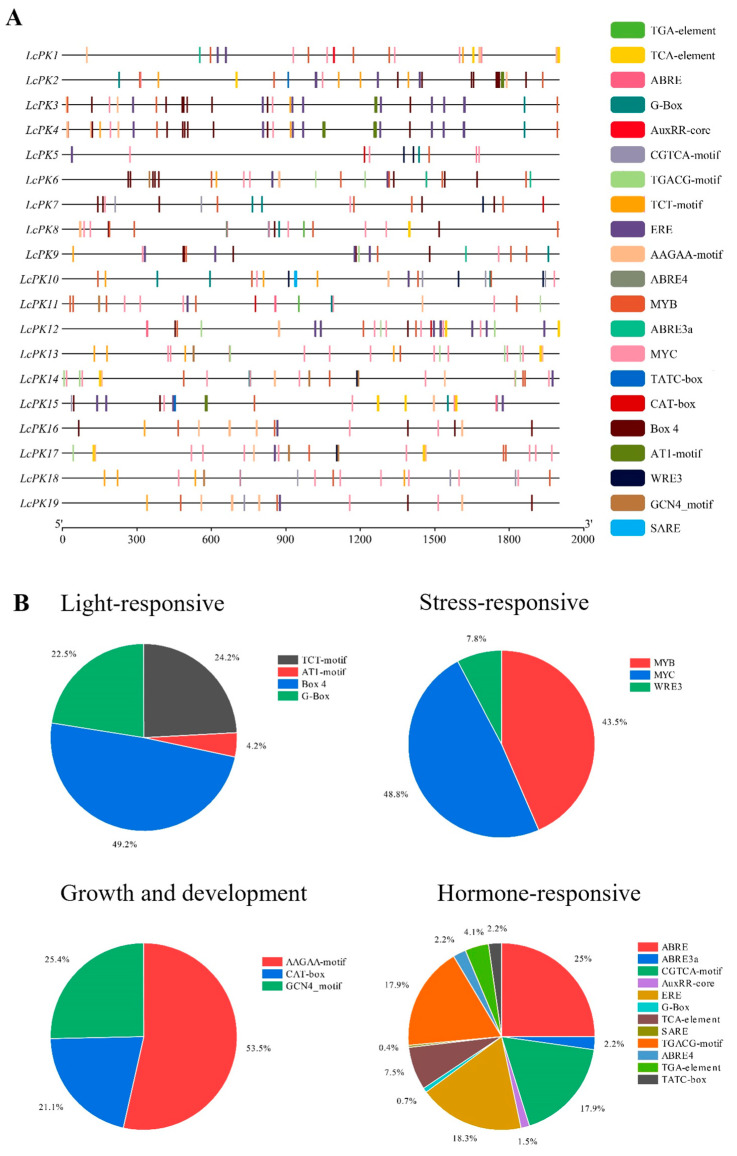
(**A**) The figure displays the cis-acting elements in the LcPK promoter, with altered colors representing each element and its respective proportions. (**B**) The pie charts showed the percentage of each cis-element in each group.

Promoter activity is essential for controlling gene expression and other biological processes. In this study, 19 *LcPK* genes were identified from the *L. chinensis* genome, and their promoter regions, defined as the 2000 base pairs upstream of the start codon, were analyzed using the PlantCare database to explore potential regulatory mechanisms and functions. The identified cis-elements suggest that *LcPK* genes may influence litchi growth, development, and responses to biotic and abiotic stresses. The total cis-acting regulatory elements were identified and classified into four groups: light-responsive, stress-responsive, growth and development-related, and hormone-responsive elements (Figure 7). In the light-responsive group, Box 4 (49.2%) was the most abundant, followed by TCT-motif (24.2%), G-Box (22.5%), and AT1-motif (4.2%). Stress-responsive elements consisted mainly of MYC (48.8%), MYB (43.5%), and WRE3 (7.8%). Growth and development-related elements were dominated by the AAGAA-motif (53.5%), with smaller proportions of GCN4 (25.4%) and CAT-box (21.1%). Within the hormone-responsive group, ABRE (25%) was most abundant, followed by ERE (18.3%) and CGTCA/TGACG-motif (17.9%), along with other elements such as ABRE3a, ABRE4, AuxRR-core, TGA-element, TATC-box, TCA-element, and SARE. These findings suggest that multiple hormone signaling pathways (ABA, GA, ethylene, MeJA, IAA, and SA) regulate *LcPK* genes, linking them to hormone fluctuations and fruit development in litchi.

### 3.6. Expression Patterns of LcPK Genes

**Figure 8:**
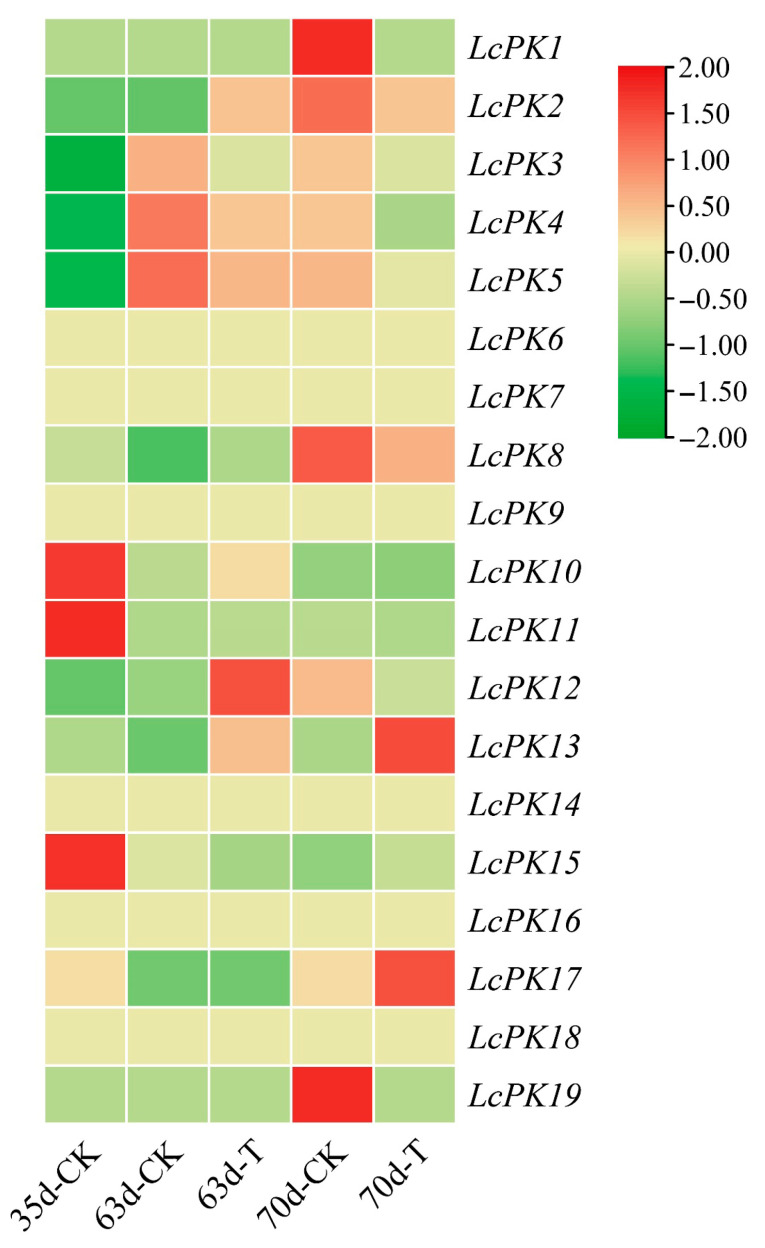
Expression patterns of *LcPK* genes under Ca+Mg treatment and CK at different time intervals. The red color in the expression bar indicates a high expression level, while the green indicates a low expression.

To investigate the effects of Ca+Mg treatment on litchi pulp, we identified 19 *LcPK* genes from the PK gene family and analyzed their expression using transcriptome data. A heatmap illustrating the expression patterns was generated with TBtools-II (Figure 8). Among these, 13 genes revealed significant expression levels based on FPKM values. The expression levels of these genes were compared between Ca+Mg-treated samples and CK during various periods. On the 63 DAA, 6 genes showed an upregulation trend, whereas 7 were downregulated under Ca+Mg treatment. By the end of the experiment, on the 70 DAA, 4 genes exhibited upregulation, while 9 genes were downregulated. The downregulation of some *LcPK* genes may aid in alleviating sugar receding in ‘Feizixiao’ litchi pulp.

### 3.7. Regulation of LcPK Genes by Phytohormones During Litchi Ripening

**Figure 9:**
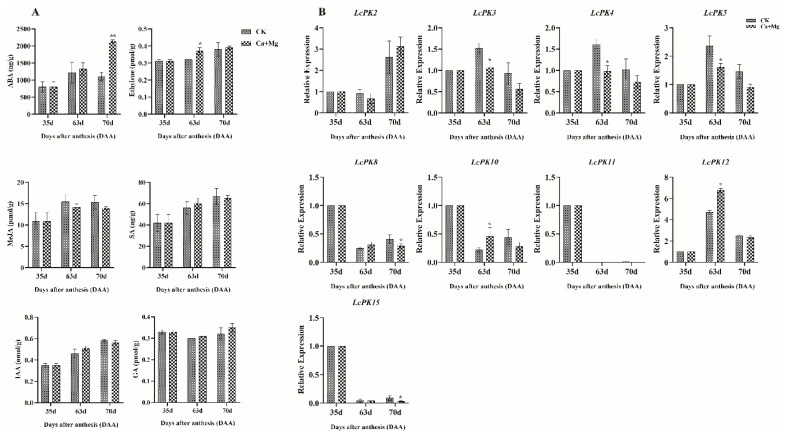
(**A**) Hormone levels (ABA, ethylene, GA_3_, MeJA, SA, IAA) and (**B**) expression of 9 *LcPK* genes in litchi pulp at 35, 63, and 70 DAA under Ca+Mg treatment and CK. Significant differences are marked * (*p* < 0.05) and ** (*p* < 0.01).

Promoter analysis of *LcPK* genes identified hormone-responsive elements linked to ABA, GA_3_, ethylene, MeJA, SA, and IAA, suggesting hormonal regulation of LcPK expression. Hormonal profiling in litchi pulp showed significant increases in ABA at 70 DAA and ethylene at 63 DAA under Ca+Mg treatment compared to CK, while MeJA, SA, GA_3_, and IAA showed no significant differences (Figure 9A). Correlation analysis between hormone levels and *LcPK* gene expression revealed distinct regulatory patterns. Among the 9 selected *LcPK* genes (LcPK2, 3, 4, 5, 8, 10, 11, 12, 15), transcript levels responded dynamically to Ca+Mg treatment across developmental stages (Figure 9B). At 63 DAA, *LcPK3*, *LcPK4*, and *LcPK5* expression were significantly downregulated, correlating with increased ethylene. At 70 DAA, *LcPK8* and *LcPK15* were also downregulated, while *LcPK10* and *LcPK12* were significantly upregulated, corresponding with peak ABA levels. *LcPK2* and *LcPK11* showed no significant change. These findings highlight a correlation between Ca+Mg-induced hormonal shifts and the differential expression of *LcPK* genes, suggesting their involvement in sugar retention, fruit development, and regulation of ripening in litchi.

## 4. Discussion

### 4.1. Effect of Ca+Mg Treatment on Sugar Content, Peel Coloration, and Metabolic Activity

Ca enhances membrane stability by binding phospholipids and reducing electrolyte leakage, while in the cell wall, it cross-links pectins to strengthen structural integrity and delay senescence [43]. Mg, as the central atom of chlorophyll and a cofactor for ATP-dependent enzymes, supports photosynthesis, carbohydrate transport, and energy metabolism [21]. Both nutrients contribute to oxidative balance: Ca participates in signaling cascades that activate antioxidant defenses [44], whereas Mg deficiency is known to increase ROS production, lipid peroxidation, and programmed cell death [45]. By stabilizing membranes and alleviating oxidative stress, Ca and Mg may create a cellular environment less prone to stress-induced transcriptional reprogramming, thereby indirectly modulating PK expression. Evidence from other fruit crops supports this interpretation. In apples, Ca treatments reduced physiological disorders such as bitter pit, maintained membrane integrity, and delayed fruit softening by limiting oxidative damage [46]. In tomato, Ca supplementation improved membrane stability and reduced fruit cracking and blossom-end rot [47]. In plants, Mg deficiency caused ROS accumulation, impaired photosynthesis, and lowered fruit quality, whereas supplementation restored redox homeostasis and antioxidant capacity [48]. These studies indicate that Ca and Mg function not only as structural and metabolic cofactors but also as modulators of cellular stability and oxidative balance. In Lychee, these roles may underlie the observed PK expression patterns by mitigating oxidative stress signaling and preserving membrane integrity during fruit development and under environmental challenges. Such indirect effects complement the direct biochemical functions of Ca and Mg, offering a broader physiological context for their regulation of PK expression. The Ca and Mg are vital for litchi growth, with calcium being crucial for cell wall stability, membrane function, and fruit development [49]. Sugar receding, a drastic drop in soluble sugar levels, reduces sweetness and spoilage of longan fruits during on-tree storage [50]. This study demonstrates that Ca+Mg treatment significantly improves the soluble sugar content and peel coloration in Feizixiao litchi, thereby enhancing fruit quality. The treatment maintained higher sugar levels at 63 and 70 DAA, preventing the “sugar receding” phenomenon, likely by stabilizing sugar content during ripening, as suggested by previous research [51]. Regarding peel coloration, the Ca+Mg treatment led to earlier and more intense reddening, accelerating the overall coloration process and resulting in a redder peel before full maturation. This aligns with studies indicating that Ca and Mg influence anthocyanin biosynthesis and chlorophyll degradation during ripening [52]. Respiration plays a vital role in energy production for fruit, driving ripening and postharvest processes through glycolysis, the TCA cycle, and the electron transport chain [53]. In our study, the Ca+Mg treatment significantly reduced the total respiration rate of Feizixiao litchi at 63 and 70 DAA compared to the CK group, indicating an inhibition of metabolic activity [54]. This suggests that Ca and Mg inhibit respiration, indicating a unique interaction between these minerals [55]. Additionally, the treatment decreased respiration through the EMP pathway from 42 to 70 DAA, with the Ca+Mg treatment showing the most substantial inhibitory effect [56]. Furthermore, the Ca+Mg treatment reduced PK activity at 49, 56, and 63 DAA, indicating inhibition of the EMP pathway during these stages. This aligns with previous studies, which have demonstrated that calcium and magnesium influence enzyme activity differently throughout fruit ripening [57,58]. Overall, the Ca+Mg treatment inhibited respiration and PK enzyme activity, thereby mitigating the “sugar receding” phenomenon in Feizixiao litchi fruit pulp.

### 4.2. Bioinformatics Analysis of PK Gene Family

The PK catalyzes the transformation of phosphoenolpyruvate (PEP) and ADP into pyruvate and ATP, a critical step in the glycolytic phase essential for energy production and metabolic regulation [59]. Gene family analysis is a fundamental and effective method for investigating gene functions [60]. We comprehensively analyzed the litchi PK gene family, including properties, 3D modeling, gene structure, phylogeny, Ka/Ks ratio, localization, synteny, cis-elements, hormone levels, and expression profiles. Bioinformatics analysis identified 19 *LcPK* genes with varying CDS lengths, protein sizes, molecular weights, isoelectric points, and GRAVY values. These characteristics exhibit varying hydrophobic and hydrophilic properties, which influence protein solubility and interactions [61,62]. The 3D structure of amino acids in random coils enhances their versatility [63]. Our study revealed that the SWISS-MODEL method predicted the 3D structures of 19 LcPK proteins with sequence identities from 30.06% to 94.69% (Figure 2). The sequence identities over 30% are reliable for homology modeling. According to previous studies, the predicted models highlight the importance of validation for accuracy [64,65].

DNA motif identification is crucial for understanding gene function, as it reveals transcription factor binding sites and the mechanisms regulating gene expression [66]. Elevated intron counts in eukaryotes promote alternative splicing, enhancing protein diversity [67]. Our analysis of conserved motifs revealed that LcPK1-5 contained all identified motifs (Figure 3a), indicating potential functional similarities [68]. These genes also possessed the conserved PK superfamily domain (Figure 3b), consistent with previous findings in *Triticum aestivum* PKs [69]. Additionally, the exon-intron structure of these genes showed a higher proportion of exons than introns (Figure 3c), suggesting a conserved structural organization and close functional relationships [70]. This finding aligns with rice PKs, suggesting PK genes evolved by modifying intron length and number to adapt to environmental changes while preserving conserved domains [71]. Phylogenetic trees are branching diagrams illustrating the historical relationships among genes or species [72]. These relationships are typically inferred using morphological characteristics, behavioral traits, or molecular sequences. The phylogenetic analysis of 59 PK proteins from *L. chinensis*, *A. thaliana*, *S. lycopersicum*, and *Malus × domestica* identified conserved and species-specific evolutionary patterns (Figure 3e). Group V highlighted functional conservation between LcPK15 and AtPK10. Moreover, Group VI demonstrated conserved relationships, notably between LcPK8 and SlPK7. Groups I and III, specific to litchi and *Arabidopsis*, respectively, suggested species-specific divergence. In contrast, Group IV revealed functional similarities across species despite overall diversity, and Group VII indicated an evolutionary link between LcPK9 and AtPK4. It is noteworthy that some *LcPK* genes did not cluster consistently between motif composition (Figure 3a) and phylogenetic grouping (Figure 3e). This discrepancy may be due to gain, loss, or rearrangement of specific motifs during evolution, reflecting functional diversification among gene family members. Such variation suggests that while *LcPK* genes share conserved sequences, certain members may have undergone sub-functionalization or neofunctionalization, leading to distinct regulatory or metabolic roles. These results emphasize the interplay between functional conservation and evolutionary divergence in the development of plant PK proteins.

Duplication events drive gene amplification, with Ka/Ks ratios revealing the types of selection [73,74]. Ka/Ks < 1 indicates purifying selection, =1 neutral, and >1 positive selection. Most LcPK gene pairs showed purifying selection, but 12 pairs indicated positive selection, similar to *Brassica rapa* [75], suggesting PK gene expansion in plants is influenced by segmental duplications. The chromosomal location analysis of the gene predicted that *LcPK* genes were unevenly distributed across the 15 litchi chromosomes, with Chr2 containing six genes (Figure 4). Similar distributions were observed in *Arabidopsis* [76]. LcPK genes are primarily localized in the cytoplasm, aligning with pyruvate kinase’s role in catalyzing glycolysis’s final step [77]. Some LcPK proteins are found in mitochondria and chloroplasts, suggesting roles in organelle-specific processes such as respiration, photosynthesis, or stress responses. This functional diversification of PK localization is also reported in *A. thaliana* and *O. sativa* [71,78,79]. The synteny analysis revealed that *N. lappaceum* has the most co-linear *LcPK* genes, followed by *D. longan* and *A. yangbiense* (Figure 6). However, *Arabidopsis* had the least common *LcPK* genes, indicating a more significant evolutionary difference between herbaceous and woody plant species. These findings suggest that the PK gene family has exhibited greater diversity in woody species, while it has remained more conserved in herbaceous species. Cis-acting elements regulate processes such as stress, hormone regulation, and development (Figure 7). In *LcPK* genes, hormone-responsive elements (e.g., ABRE, ERE, TGA, AuxRR-core) indicate hormone influence, particularly during fruit development and stress, similar to other species like grapes and tomatoes [80,81]. This suggests a potential role for *LcPK* genes in the hormone-nutrient interaction network that regulates key developmental and metabolic processes during fruit maturation.

### 4.3. Correlation Between Hormonal Regulation and LcPK Gene Expression Analysis Overcomes Sugar-Receding Process

Phytohormones such as ABA, ethylene, GA, MeJA, IAA, and SA are essential regulators of fruit development, ripening, and stress responses [82]. Our promoter analysis of *LcPK* genes revealed the presence of multiple hormone-responsive elements, indicating that hormonal signals likely regulate these genes. Hormonal profiling demonstrated that ABA levels significantly increased at 70 DAA, and ethylene peaked at 63 DAA under Ca+Mg treatment (Figure 9). Litchi is classified as a non-climacteric fruit, meaning it does not undergo a sharp increase in ethylene production or respiration during ripening, unlike climacteric fruits such as tomatoes or bananas [83,84]. Our study found that ABA levels were significantly higher in the Ca+Mg treatment than in the CK at 70 DAA, supporting the idea that ABA plays a key role in litchi fruit maturation. This is consistent with studies showing that ABA regulates critical ripening processes in non-climacteric fruits, including anthocyanin accumulation and chlorophyll breakdown [85,86,87]. Although ethylene is the primary ripening hormone in climacteric fruits, its role is limited in non-climacteric fruits like litchi. In our results, ethylene levels increased significantly at 63 DAA but decreased as the fruit matured, particularly in the pulp. This trend has been reported in earlier litchi studies, where ethylene was found to be more active in young fruit, particularly in the pericarp [86]. While ethylene may contribute to pigment development by promoting anthocyanin synthesis, it does not trigger ripening in litchi as in climacteric fruits. These findings confirm that ABA is the primary hormone-regulating litchi ripening, while ethylene plays a secondary or tissue-specific role. This hormonal behavior is typical of other non-climacteric fruits, such as strawberries and grapes, where ABA initiates and drives ripening processes [85]. Therefore, our results support that litchi ripening is mainly ABA-dependent, and targeted postharvest strategies should consider this hormonal regulation. Expression analysis of nine *LcPK* genes showed diverse responses to Ca+Mg treatment and associated hormonal fluctuations. Specifically, *LcPK3*, *4*, *5*, *8*, and *15* were downregulated, whereas *LcPK10* and *12* were upregulated during key developmental stages. Protein kinase (PK) gene families often show different expression patterns, and in many cases, more than one member can perform similar roles. For example, in *Arabidopsis*, three SnRK2 protein kinases (SRK2D/E/I) work together in ABA-induced leaf senescence, and only when all three are knocked out is the effect seen, showing partial functional redundancy [88]. To properly understand the hormonal role of PKs, correlation studies alone are not enough. Functional validation using treatments with ABA or ethylene, or their inhibitors, is important. For instance, applying ABA to detached *Arabidopsis* leaves triggers senescence in wild-type plants but not in the SRK2D/E/I triple mutant [89]. Similarly, transcriptome studies of ABA and ethylene mutants under stress show clear crosstalk between these hormones, supporting the value of exogenous treatments and inhibitor studies [90]. The PK is a glycolysis rate-limiting enzyme that converts phosphoenol pyruvate to pyruvate while producing ATP [91]. The downregulation of several *LcPK* genes suggests a reduction in glycolytic activity, which could slow sugar catabolism and favor sugar retention in fruit pulp. This may contribute to overcoming the sugar-receding phenomenon commonly observed in litchi. Additionally, reduced glycolytic flux might influence other metabolic processes such as acid metabolism and cell wall modification, thereby affecting fruit texture and ripening progression [92,93,94,95]. These findings suggest that Ca+Mg treatment alters hormone levels, which in turn modulate the expression of *LcPK* genes, linking hormonal regulation to metabolic adjustments that enhance sugar retention and improve fruit quality during litchi ripening.

## 5. Conclusions

The Ca+Mg treatment prevented “sugar receding” and improved peel coloration in ‘Feizixiao’ litchi by increasing sugar content, accelerating reddening, and inhibiting respiration and PK enzyme activity, thereby enhancing fruit quality. We also analyzed the litchi PK gene family, identifying 19 LcPK members and investigating their structural and functional characteristics. Our results revealed variation in physical properties, conserved motifs, domains, and gene structures, highlighting functional conservation. Phylogenetic analysis across plant species indicated both conserved functions and species-specific divergence, offering insights into the evolutionary adaptation of PK proteins in signaling and metabolism. Collinearity between *A. thaliana* and related species further explained LcPK expansion. The identification of multiple cis-acting elements associated with development and hormone signaling suggested a complex regulatory network. Differential expression patterns of *LcPK* genes at 63 and 70 DAA, along with changes in ABA and ethylene, indicated their regulatory roles in sugar metabolism. Under Ca+Mg treatment, elevated hormone levels and downregulation of LcPK expression may help overcome “sugar receding” in fruit pulp. Overall, this study highlights the importance of the PK gene family in litchi fruit quality and development and provides a foundation for functional characterization to further improve ‘Feizixiao’ fruit quality.

## Figures and Tables

**Table 1:** LcPK genes’ physical and biological features.

Transcript ID	Given Name	Chr.	Strand	CDS (bp)	PL (A.A)	PMW (kDa)	pI	GRAVY
LITCHI024313.m1	*LcPK1*	13	Forward	1512	503	54.61	6.73	−0.023
LITCHI003418.m1	*LcPK2*	6	Forward	1542	513	55.35	6.35	0.070
LITCHI006026.m1	*LcPK3*	14	Reverse	1815	604	65.68	8.45	0.072
LITCHI006031.m1	*LcPK4*	14	Reverse	2130	709	77.42	6.83	−0.032
LITCHI018757.m1	*LcPK5*	15	Reverse	1584	527	57.47	7.14	0.026
LITCHI020380.m1	*LcPK6*	12	Forward	1698	565	62.30	6.66	−0.174
LITCHI027188.m1	*LcPK7*	3	Forward	1353	450	49.32	6.77	−0.008
LITCHI009466.m1	*LcPK8*	7	Forward	1737	578	63.98	6.34	−0.210
LITCHI014910.m1	*LcPK9*	1	Forward	1755	584	64.08	5.47	−0.101
LITCHI026147.m1	*LcPK10*	3	Reverse	1557	518	57.26	5.59	0.025
LITCHI001406.m1	*LcPK11*	5	Forward	1755	584	64.84	5.93	−0.141
LITCHI008504.m1	*LcPK12*	7	Reverse	237	78	86.10	8.69	−0.262
LITCHI012288.m1	*LcPK13*	2	Reverse	1254	417	46.75	6.08	−0.313
LITCHI012309.m1	*LcPK14*	2	Forward	693	230	25.77	5.43	−0.174
LITCHI010067.m1	*LcPK15*	8	Forward	2106	702	77.50	7.84	−0.172
LITCHI012284.m1	*LcPK16*	2	Forward	576	191	21.51	7.65	−0.175
LITCHI012308.m1	*LcPK17*	2	Forward	849	282	31.85	6.41	−0.350
LITCHI012302.m1	*LcPK18*	2	Reverse	324	107	12.00	5.00	0.036
LITCHI012287.m1	*LcPK19*	2	Reverse	951	316	35.62	5.06	−0.198

## Data Availability

The original contributions presented in this study are included in the article/Supplementary Material. Further inquiries can be directed to the corresponding author.

## Supplementary Materials

The following supporting information can be downloaded at: https://www.mdpi.com/article/10.3390/plants14172764/s1. Table S1. Primer table of *LcPK* genes. Table S2. Ka and Ks substitution rates of *LcPKs* gene pairs. Table S3. Subcellular localization prediction of *LcPK* genes.
